# A Case of Female Acute Urinary Retention Presenting to the ED

**DOI:** 10.1155/2017/4598314

**Published:** 2017-08-16

**Authors:** Kylen Swartzberg, Ahmed Adam, Feroza Motara, Abdullah E. Laher

**Affiliations:** ^1^Department of Emergency Medicine, Charlotte Maxeke Johannesburg Academic Hospital and University of the Witwatersrand, Faculty of Health Sciences, Johannesburg, South Africa; ^2^Department of Urology, Helen Joseph Hospital, Johannesburg, South Africa; ^3^Department of Paediatric Urology, Rahima Moosa Mother & Child Hospital, Johannesburg, South Africa; ^4^Division of Urology, Department of Surgery, Faculty of Health Sciences, University of the Witwatersrand, Johannesburg, South Africa

## Abstract

**Introduction:**

Acute urinary retention is a rare occurrence in women necessitating further investigation. Potential underlying causes may be broadly classified into obstructive, neurological, pharmacological, and psychogenic categories.

**Case:**

A 36-year-old nulliparous female presented to the Emergency Department with a two-day history of acute urinary retention. Point-of-care ultrasonography and CT scan imaging confirmed the presence of a large uterine mass causing compression of the bladder. The acute retention was relieved with urethral catheterization. A Uterine leiomyoma was confirmed on histology after hysterectomy.

**Discussion:**

Once the acute urinary retention has been relieved by insertion of a urethral catheter, the underlying cause of the obstruction must be determined. Although uterine leiomyoma is a fairly common finding in the general population, it is an extremely rare cause of acute urinary retention in women with just a handful of reported cases in the literature.

## 1. Introduction

Urinary retention in women is a rare occurrence, with an overall incidence of 0.07 per 1000 inhabitants per year [1]. Herein we describe our experience with a young female patient who presented to the Emergency Department [ED] with acute urinary retention.

## 2. Case Report

**Figure 1:**
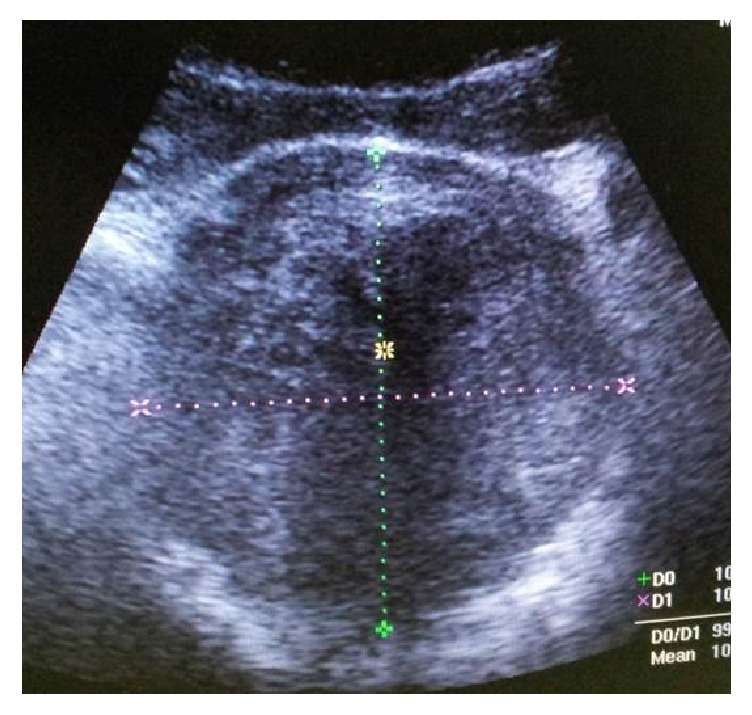
Abdominopelvic ultrasound study showing a well-defined uterine mass measuring 10,5 cm × 10,6 cm.

**Figure 2:**
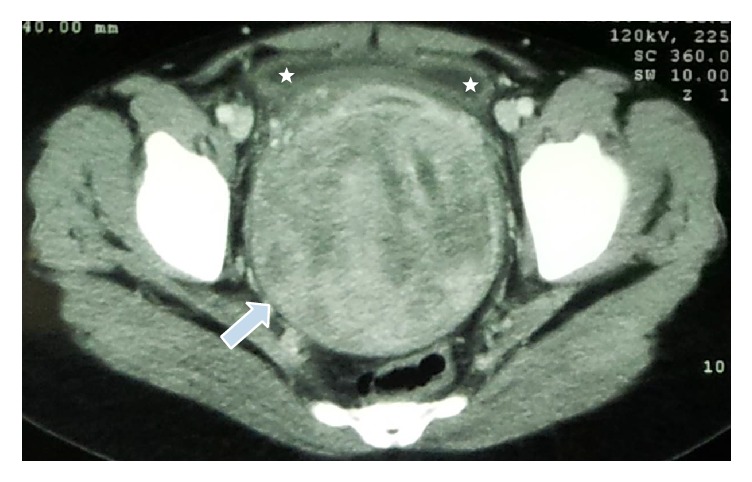
Pelvic CT scan outlining the uterine lesion (white arrow). The compressed bladder lumen is also illustrated (white stars).

A 36-year-old nulliparous female presented to the ED with a history of severe lower abdominal pain and an inability to pass urine for the last 8 hours. Abdominal examination revealed a tender palpable bladder midway between the pubic symphysis and umbilicus. The rest of the clinical assessment including medication history, gynaecological examination, and neurological assessment was unremarkable. Serum electrolytes, urea, creatinine, and calcium were all within normal limits. A large distended bladder as well as a pelvic mass was visualised on point-of-care ultrasonography (Figure 1). An abdominal CT scan that was requested after insertion of a size 14 French urinary catheter reported the presence of a large posterior uterine wall mass (10,5 cm × 10,6 cm), anterior displacement of the urinary bladder, and mild (grade I) bilateral hydronephrosis/hydroureter (Figure 2). After being transferred to the gynaecology ward, she later underwent a total abdominal hysterectomy where after she was discharged with no residual urinary symptoms. Histology confirmed a uterine leiomyoma (fibroid) as the cause of the obstruction.

## 3. Discussion

Female urinary retention is a rare occurrence. The underlying aetiology can be divided into the following four categories: neurological, obstructive, pharmacological, and psychogenic [2].

Uterine leiomyoma is the commonest benign gynaecologic tumour with up to one in four women developing one or more fibroids during their lifetime. Uterine fibroids occur more frequently in women aged 30–50 years and is strongly associated with obesity, nulliparity, and a positive family history [3, 4]. Despite it being so common, uterine leiomyoma is an extremely rare cause of acute urinary retention in women with just a handful of reported cases in the literature [5–8]. In a case series that included 8 women who presented to the ED with acute urinary retention, seven (88%) of these patients had posterior-fundal based leiomyomas. The authors postulated that incarceration of a posterior-fundal based leiomyoma beyond the pelvic brim resulted in compression of the bladder neck or urethra by displacing the cervix against the pelvic bone [3]. Yang and Huang reported their ultrasound findings of six patients with acute urinary retention secondary to an impacted pelvic mass. They concluded that obstruction of the internal urethral orifice was as a result of compression of the lower bladder from displacement of the cervix and not due to compression or distortion of the urethra itself [8]. Another case report demonstrated electromyographic evidence of pelvic plexus compression by a uterine fibroid resulting in acute urinary retention [9]. Other pathophysiologic theories explaining urinary retention secondary to uterine leiomyoma include the* “vascular steal effect*,*”* where blood is shunted away from the urogenital system resulting in detrusor muscle hypocontractility and urinary retention as a consequence. Based on this theory, Arleo and Tal reported successful resolution of fibroid induced acute urinary retention after uterine artery embolization [10].

Other obstructive causes of urinary retention include; cervical cancer, urethral stenosis, urethral cancer and anti-incontinence surgery [11, 12]. A recent South African review, reporting on 31 women presenting with urinary retention over a 9-year period, identified urethral carcinoma (4/31) and cervical carcinoma (4/31) as the commonest underlying pathology [13].

Overall, neurologic conditions (e.g., Fowlers syndrome, spinal cord-compression, and multiple sclerosis) make up the most common causes of acute urinary retention in young to middle-aged women [11, 12]. In a four-year review of cases presenting to their centre, Kavia and colleagues reported Fowlers syndrome (poor relaxation of the external urethral sphincter) as the most common cause of female urinary retention. This condition is strongly associated with polycystic ovarian syndrome and opiate use in young women [14, 15].

Pharmacologic agents often implicated as the cause of acute urinary retention include tricyclic antidepressants, opioid analgesics, antipsychotics, antimuscarinics, and *α*-adrenergic agonists [16]. The diagnosis of an underlying psychogenic aetiology as a cause of urinary retention is usually one of exclusion after a thorough investigative workup [17].

Once urinary retention is suspected or confirmed with point-of-care ultrasonography when available, bladder catheterization to relieve patient distress takes priority prior to conducting further investigations that may include computed tomography scanning, magnetic resonance imaging, and electromyography [12].

## 4. Conclusion

Female urinary retention is a rare entity and its underlying origin may pose a management dilemma. Early catheterization to relieve patient distress must always precede a search for the underlying aetiology and associated complications.
